# Advances in Tunable Bandgaps of Piezoelectric Phononic Crystals

**DOI:** 10.3390/ma16186285

**Published:** 2023-09-19

**Authors:** Yiwei Wang, Xiaomei Xu, Li Li

**Affiliations:** College of Automobile and Traffic Engineering, Nanjing Forestry University, Nanjing 210037, China

**Keywords:** phononic crystal, piezoelectric ceramics, bandgap characteristics, tunable bandgap

## Abstract

Bandgaps of traditional phononic crystals (PCs) are determined using structural geometric parameters and material properties, and they are difficult to tune in practical applications. Piezoelectric PCs with lead zirconium titanate piezoelectric ceramics (abbreviated to piezoelectric PCs) have multi-physics coupling effects and their bandgaps can be tuned through external circuits to expand the application range of the PCs. First, the typical structures of piezoelectric PCs are summarized and analyzed. According to the structure, common tunable piezoelectric PCs can be roughly divided into three categories: PCs that only contain piezoelectric materials (single piezoelectric PCs), PCs composed of embedded piezoelectric materials in elastic materials (composite piezoelectric PCs), and PCs that are composed of an elastic base structure and attached piezoelectric patches (patch-type piezoelectric PCs). Second, the tuning methods of bandgaps for piezoelectric PCs are summarized and analyzed. Then, the calculation methods of the bandgaps of piezoelectric PCs are reviewed and analyzed. Finally, conclusions are drawn on the research status of piezoelectric PCs, shortcomings of the existing research are discussed, and future development directions are proposed.

## 1. Introduction

Phononic crystals (PCs) are periodic composite materials with lattice spacing comparable to the acoustic wavelength. They are of interest because of the profound effects of their periodic structure on wave propagation (e.g., the existence of acoustic bandgaps) and potential applications (e.g., their possible role in sound filters, transducer design, and acoustic lenses) [1]. Kushwaha et al. [2] first proposed the concept of PCs and noted their prospective applications in controlling the vibration of high-precision mechanical equipment. The main feature of PCs is that the propagation of elastic waves is restrained or blocked within a certain frequency range, which is called the bandgap. The major mechanisms of bandgap generation for PCs include Bragg scattering, local resonance (LR), level repulsion, and inertial amplification [3]. The generation of Bragg bandgaps in PCs is primarily influenced by the interaction among different structural units. Liu et al. [4] elucidated the mechanism of local resonant (LR) bandgaps in PCs. Unlike Bragg scattering, the local resonant bandgap (LRG) does not necessitate significant structural periodicity. Instead, it primarily relies on the characteristics of individual scatterers. When two bands with the same polarization intersect, hybridization bandgaps may emerge due to level repulsion. By incorporating the inertial amplification mechanism, wide bandgaps at low frequencies can be achieved. Other mechanisms have also been reported, such as wavenumber bandgaps [5,6] and amplitude bandgaps [7].

The regulation of elastic waves in PCs is determined by their structural geometric parameters and material properties, which are often fixed and cannot be easily changed in practical applications. As a result, the practical application of PCs is greatly limited. However, the pre-existing state of stress in a PC, also known as the “prestress”, can have a strong influence on the propagation of waves in elastic structures. This effect provides a potential means to alter the characteristics of elastic waves by adjusting the prestress. Nevertheless, both experimental [8] and numerical [9,10] investigations have indicated that significant tunability of bandgaps in traditional hard materials requires the application of exceptionally large prestresses. This requirement makes the manipulation more difficult and introduces other problems concerning energy consumption and structural safety. Soft materials and granular systems are good candidates for designing tunable PCs using prestress. Goffaux et al. [11] initiated research on tunable PCs and discovered that rotating the non-axisymmetric solid scatterers in PCs at a certain angle could tune the bandgap width of the structure. Subsequent studies of tunable PCs demonstrated that tuning the bandgap mainly involved the use of exquisite asymmetric PC structures and the introduction of smart materials in PCs. The latter commonly uses multi-field-coupled materials, and their bandgaps are tuned using the sensitivity of their material properties to external stimuli, such as external mechanical excitations [12,13,14], temperature field excitations [15], magnetic field excitations [16], and electric field excitations [17,18,19,20].

Piezoelectric materials play a unique role in the research and design of tunable PCs and possess significant application value [21,22,23]. These materials belong to the category of intelligent or smart materials, characterized by their ability to couple multiple physical fields. In piezoelectric materials, mechanical energy and electrical energy can be mutually converted. There are several types of piezoelectric materials, such as piezoelectric elastomers, polyvinylidene fluoride, and piezoelectric ceramics (e.g., lead zirconium titanate, PZT). Among them, piezoelectric ceramics have a high piezoelectric constant, well-established manufacturing processes, low cost, and a wide range of applications. Consequently, this study primarily focuses on the research status and future prospects of PCs incorporating piezoelectric ceramics.

This study reviews the advances in the tunable bandgaps of piezoelectric PCs containing piezoelectric ceramics. The remainder of the paper is organized as follows. Section 2 summarizes and demonstrates the structures of piezoelectric PCs. Section 3 reviews the tuning methods of the bandgaps of piezoelectric PCs. Section 4 summarizes the calculation methods of bandgaps. Section 5 provides concluding remarks and the outlook.

## 2. Structures of Piezoelectric Phononic Crystals

According to the structure, common tunable piezoelectric PCs can be roughly divided into three categories: PCs that only contain piezoelectric materials (single piezoelectric PCs), PC composed of embedded piezoelectric materials in elastic materials (composite piezoelectric PCs), and PCs that are composed of an elastic base structure and attached piezoelectric patches (patch-type piezoelectric PCs).

### 2.1. Single Piezoelectric PCs

Single piezoelectric PCs are a type of piezoelectric PC that utilizes a single homogeneous piezoelectric material. These PCs achieve their specific functionalities by varying the structural geometric parameters, such as thickness or cross-section size, and by incorporating periodic hole arrangements with embedded or attached periodic electrodes [24]. The periodic piezoelectric patches can create bandgaps [25], but these generated bandgaps are typically narrow and offer limited adjustability [26]. In the single piezoelectric PCs discussed in this section, the piezoelectric materials are typically connected to external circuits through periodically arranged electrodes. The bandgap primarily emerges due to the periodic electrical boundary conditions imposed on the piezoelectric material. The reported structures include one-dimensional (1D) rod-type piezoelectric PCs and two-dimensional (2D) piezoelectric PC plates.

**Figure 1:**
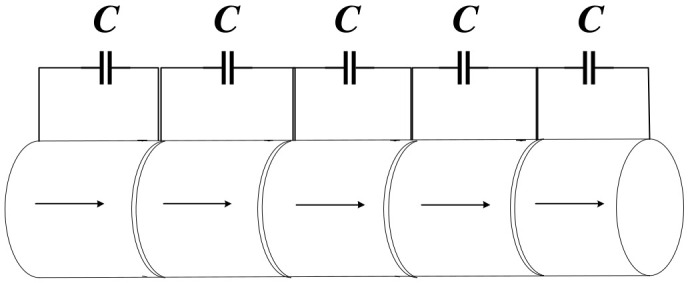
Schematic of the 1D rod-type piezoelectric PC. Reprinted with permission from Ref. [27]. 2014, Degraeve, S.

**Figure 2:**
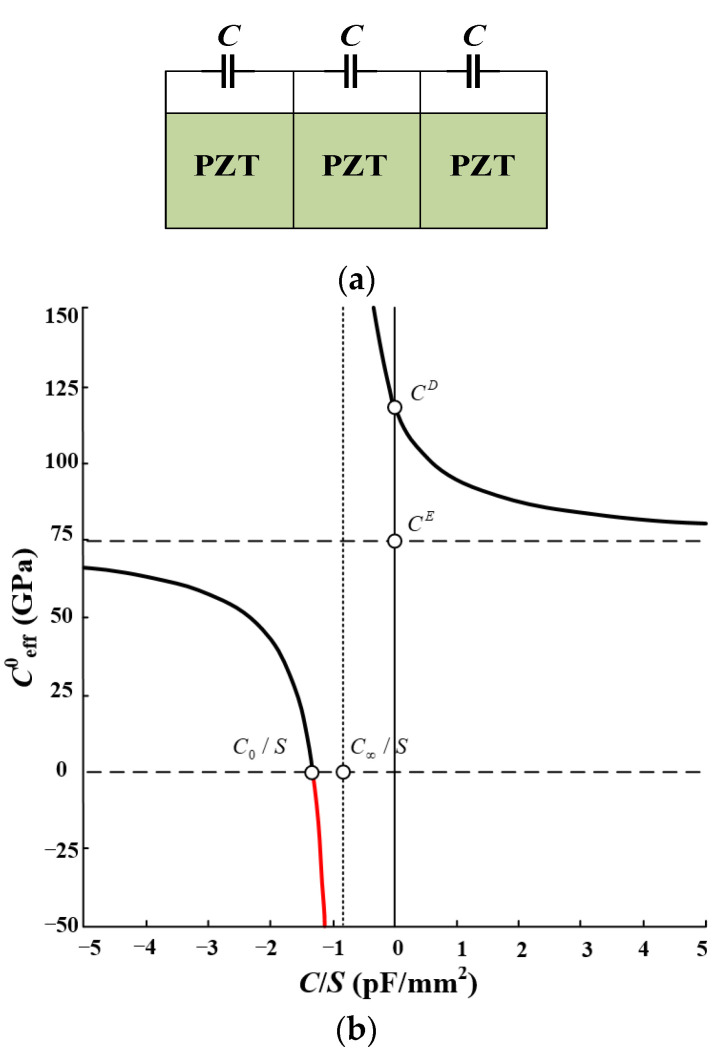
PZT periodically layered piezoelectric structure and the relationship between the parameters. (**a**) Structure of the piezoelectric PC; (**b**) dependence of the quasistatic effective elastic constant on the external capacitance C [30].

Tuning the Bragg bandgaps through periodic electrical boundary conditions was first proposed by Degraeve et al. [27]. They constructed a 1D uniformly laminated rod-type piezoelectric PC, and Figure 1 shows the structure. Studies have demonstrated that the generated bandgaps can be tuned by external capacitors. When there is a short circuit (SC) between adjacent electrodes, the maximum bandgap can be obtained. When there is an open or broken circuit between adjacent electrodes, the bandgap will disappear. In addition, the bandgap of the rod-type piezoelectric PC can be diversified more by changing the external circuits [28]. Currently, the external circuits of this type of piezoelectric PC are mostly simple. Kutsenko et al. [29] studied a complex external circuit of the piezoelec-tric PC and found many novel and very interesting physical phenomena. The normal propagation of the longitudinal wave through the piezoelectric medium with periodically embedded electrodes was studied [30]. In the PZT periodically layered piezoelectric structure, each pair of electrodes was connected via a circuit with the capacitance (C). The unusual features of the dispersion spectrum that arise in the special case of a negative capacitance (NC) were analyzed in detail. Figure 2b shows the dependence of the quasistatic effective elastic constant $c_{\mathrm{eff}\;}^{0}\;$ on the external capacitance *C* for the PZT periodically layered structure depicted in Figure 2a. The value $c_{\mathrm{eff}\;}^{0}$ is negative for $\;C/S\in \left(C_{0}/S,C_{\infty }/S\;\right)$ where $C_{0}/S$≈ −1.3295 pF/mm^2^, $C_{\infty }/S$ = −0.833 pF/mm^2^. The 1D rod-type piezoelectric PC is not only simple in structure but also highly tunable in terms of bandgap. This makes it a valuable tool for designing new elastic wave devices and exploring novel elastic wave phenomena. One potential application is the design of spatio-temporal phononic crystals, which can be achieved by utilizing time-varying electrical boundary conditions. Additionally, these structures can be utilized to design electrically tunable elastic wave topologies, opening up further possibilities for controlling and manipulating elastic waves.

**Figure 3:**
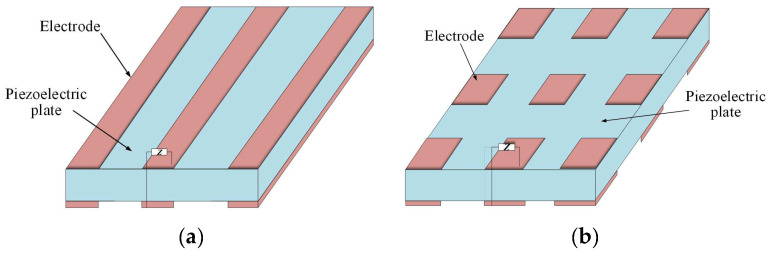
Schematics of 2D single piezoelectric PCs (**a**) The 2D piezoelectric plate with periodic strip electrodes. (**b**) The 2D piezoelectric plate with periodic rectangular electrodes.

**Figure 4:**
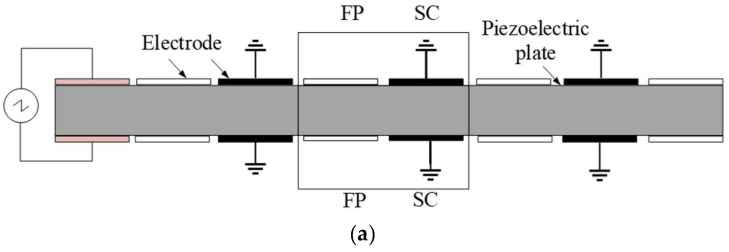

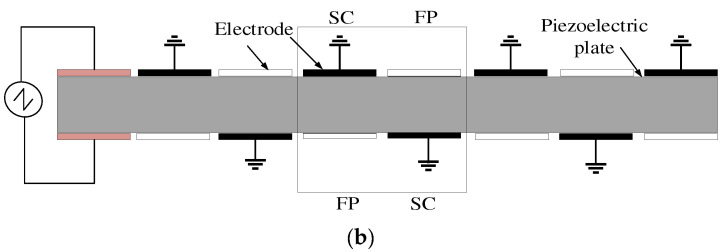
Piezoelectric PCs with (**a**) symmetric and (**b**) asymmetric electrical boundary conditions. Reprinted with permission from Ref. [32]. 2016, Kherraz, N.

Two-dimensional piezoelectric PCs are mainly based on a piezoelectric pole or plate [31] whose surface is coated or pasted with a periodic electrode, which is connected to the external circuits with equivalent impedance Z, as shown in Figure 3. Kherraz et al. [32] proposed a 2D piezoelectric PCs plate, as shown in Figure 4. They creatively simulated the active control of acoustic waveguides under symmetrical and asymmetric electrical boundary conditions (such as the floating potential, SC, and when connected to the ground). Then, they used a similar structure to tune the propagation of the elastic-guided wave by connecting the inductance load to the electrode array [26]. The results indicate that the bandgap of the structure is related to the inductance value (L) and the size and mode of the conductive electrode distribution along the plate.

### 2.2. Composite Piezoelectric PCs

**Figure 5:**
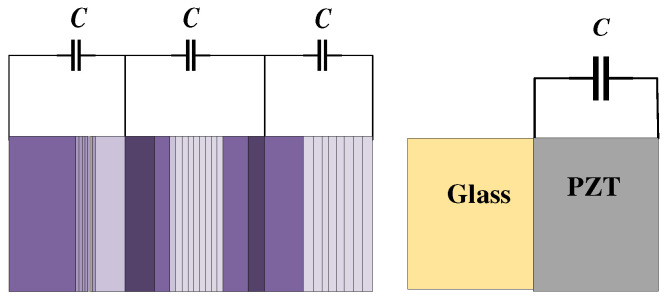
Schematics of different elastic-piezoelectric multilayer units. Reprinted with permission from Ref. [25]. 2015, Kutsenko, A.A.

**Figure 6:**
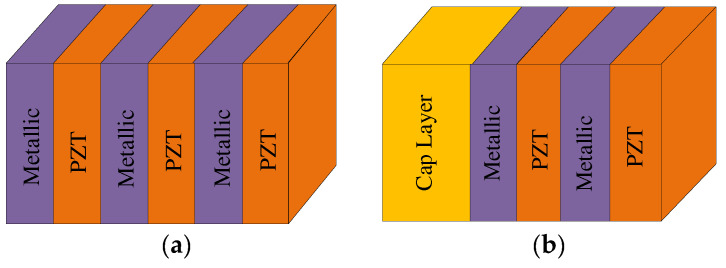
Schematics of (**a**) an infinite and (**b**) a semi-infinite piezoelectric/metallic SL. Reprinted with permission from Ref. [33]. 2018, Alami, M.

Composite piezoelectric PCs consist of piezoelectric materials embedded in elastic materials. The embedded piezoelectric materials include layers and columns. The study of layered structures mainly includes the effective properties of the piezoelectric multilayer and the influence of the cap layer. Kutsenko et al. [25] studied the general case, i.e., the unit shown in Figure 5, which is composed of random elastic-piezoelectric multilayers (possibly functional gradients) that are separated by connected electrodes. The results provide the possibility to model the effective properties of more complicated periodic multilayers. Alami et al. [33] proposed 1D infinite and semi-infinite piezoelectric superlattices (SLs), as shown in Figure 6. The existence mechanism of two complementary surface modes of semi-infinite SLs can be obtained via the cleavage of infinite SLs along a plane parallel to the piezoelectric layers. The surface modes of semi-infinite SLs are related to the open circuit and SC of the cap layers. In a semi-infinite SL, the electric field is changed by covering the piezoelectric cap layer on the surface of the piezoelectric layer. The four electrical boundary conditions of the piezoelectric layer are applied or switched to the piezoelectric layer: electric opening, applied capacitor, electric SC, and applied feedback voltage. The results show that the structure may have an interfacial mode and can interact with the Bleustein-Gulyaev surface mode in the same bandgap. The strength of the interaction depends on the width of the cap layer. The electromechanical coupling coefficient is very sensitive to the cap layer thickness.

**Figure 7:**
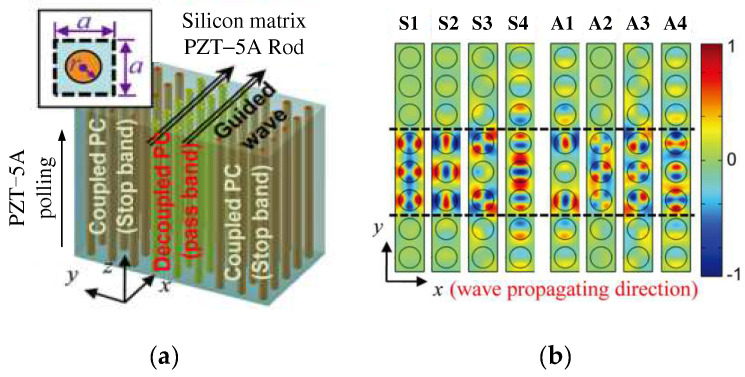
Schematics of (**a**) the PC with piezoelectric rod inclusions and (**b**) the mode shapes of the cells. Reprinted with permission from Ref. [34]. 2011, Oh, J.H.

There is another composite piezoelectric PC where the piezoelectric material acts as a columnar scatterer. Oh et al. [34] found that active wave-guiding could be realized in a stop band frequency range of a phononic crystal (PC) if piezoelectric inclusions in the PC were electrically controlled. The PC structure considered in their work has PZT - 5A rods embedded in a bulk silicon matrix shown in Figure 7a, and Figure 7b shows the mode shapes of the guided modes for the frequency range corresponding to the stop band of the coupled PC. The feasibility of this structure was confirmed using the finite element method (FEM). The propagation mechanism of the incident plane wave in the waveguide was explained using SL dispersion and wave pattern analysis. Then, Hu et al. [35] designed a 2D piezoelectric crystal structure that consisted of a square piezoelectric rod embedded in an epoxy resin matrix, where the inductor circuit independently controlled the performance of the unit. By appropriately selecting external circuit parameters, LR can be combined with complete Bragg scattering. By adjusting the L of the external circuit, the appropriate operating frequency range can be selected. This structure can be used to simultaneously suppress vibrations and harvest energy. Dwivedi et al. [36] discovered that the bandgap characteristics of piezoelectric PC with negative stiffness depended on the dimensionless capacitance (C) and resistance (R) parameters.

In summary, these two types of piezoelectric PCs generally use periodic electrodes to connect the piezoelectric material with external circuits. The bandgap is mainly generated via periodic electrical boundary conditions. With the increase in the dimensions of structures and the types of external circuits, the complexity and diversity of bandgaps are increasing. Studies on the coupling of piezoelectric PCs to complex external circuits are currently lacking. Some unique microstructures or related concepts such as resonance units have been introduced to promote an integrated design [37,38,39,40,41,42]. The topology optimization method was introduced into the design of piezoelectric PCs [43] to improve the acoustic characteristics of the structure. In addition, if the symmetry of the structure is broken, e.g., by modifying the electric field boundary conditions of the upper and lower electrodes and achieving the LRG against the Lamb wave mode, there may be a unique phenomenon.

### 2.3. Patch-Type Piezoelectric PCs

**Figure 8:**
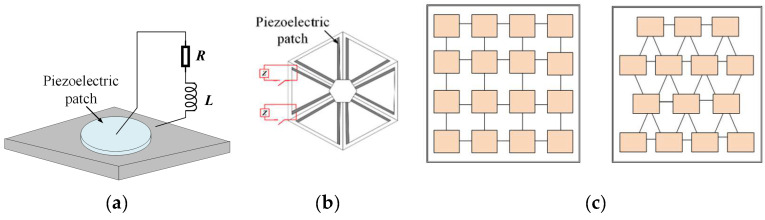
Schematics of piezoelectric PCs containing a unit with a single piezoelectric patch (**a**) Schematic model of 2D piezoelectric PCs and the resonating unit (**b**) 2D honeycomb metamaterial structures with piezoelectric patches (**c**) Two-dimensional periodic square lattice and equilateral triangle lattice. Reprinted with permission from Ref. [44]. 2011, Casadei, F. Reprinted with permission from Ref. [45]. 2015, Celli, P. Reprinted with permission from Ref. [46]. 2008, Zou, X.Y.

Patch-type piezoelectric PCs are commonly made of piezoelectric materials and formed into piezoelectric patches applied by being affixed to the elastic body as sensors or actuators. Casadei et al. [44] combined Bragg scattering with piezoelectric resonators to construct 2D piezoelectric PCs, experimentally confirmed the possibility of achieving elastic waveguides with tunable dispersion characteristics, and first proposed a novel method of using piezoelectric patches to achieve waveguides. For the same purpose, Celli et al. [45] pasted piezoelectric patches into 2D honeycomb metamaterial structures to tune the propagation of elastic waves in different directions. Zou et al. [46] discovered that the first-order bandgap of 2D patch-type piezoelectric PCs was related to factors such as the fill rate and polarization direction. Figure 8 shows the piezoelectric PC using a single piezoelectric patch. Extensive systematic research has been conducted by Baz et al. [47] on one-dimensional acoustic superstructures, where the equivalent density and stiffness are actively controlled using periodic arrangements of piezoelectric sheets within a fluidic cavity. Furthermore, significant progress has been made in utilizing piezoelectric sheets for various applications, such as actively controlled negative refraction imaging [48], adaptive gradient index (GRIN) lenses [49], and acoustic stealth cloaks [50]. For instance, Ning et al. [51,52] successfully enhanced the stealth and black hole behavior of elastic waves by attaching piezoelectric sheets with external negative capacitance circuits onto a superstructured material plate. The above research shows that this structure is relatively simple, but its tunable range of bandgaps is narrow and the flexibility is poor.

**Figure 9:**
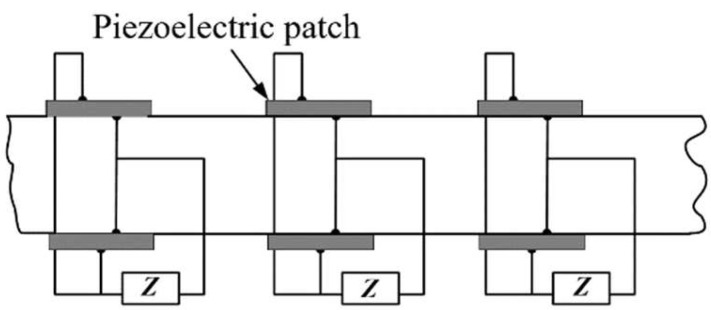
Schematic of the rod-like piezoelectric PC. Reprinted with permission from Ref. [53]. 2001, Thorp, O.

Compared with the piezoelectric PCs that use a single piezoelectric patch, the piezoelectric PCs using double piezoelectric patches are advantageous in tuning bandgaps. Piezoelectric parallel arrays are typical examples. They are powerful tools to change the equivalent mechanical impedance to control the dynamic fluctuation behavior by changing the tunable properties of parallel piezoelectric patches. Thorp et al. [53] controlled the propagation of longitudinal waves in a rod-like structure by periodically distributing parallel piezoelectric patches, as shown in Figure 9. They derived and calculated the equivalent Young’s modulus of the piezoelectric patches in the external circuit. This finding confirmed that the effect of the shunt circuit on the piezoelectric patch was to change the equivalent Young’s modulus, and this adjustment method could obtain a negative equivalent elastic modulus. Since then, several researchers, including Airoldi et al. [54], have also studied the propagation of waves in similar structures. However, 2D piezoelectric PCs have a higher dimension than the 1D structure, which introduces more vibration and wave modes. More novel, extraordinary, and unexpected vibrations and fluctuations have been discovered and studied. Chen et al. [55] proposed an effective method to predict the dispersion relationship of waves propagating in any direction of 2D piezoelectric PCs, and they studied the effects of damping and electrical resonance on the propagation of waves in all directions. Studies have demonstrated that the resistive shunt can adjust the position of the bandgap and attenuation constant. In addition, the internal resonance of the resonant shunt system splits the dispersion curve to form LRGs. LRGs in piezoelectric PCs have attracted the attention of researchers. Casadei et al. [56] demonstrated that connecting an array of active NC shunt circuits and a passive parallel resonant shunt circuit to different piezoelectric chip arrays can effectively widen the bandgap. Subsequently, Zhang et al. [57] constructed bandgaps based on a periodic array of piezoelectric patches based on mixed shunts. An active shunt circuit and a passive resonant shunt circuit are connected parallel to the same piezoelectric patch. Studies have shown that wider bandgaps can be obtained when LRGs are coupled to bandgaps. 

**Figure 10:**
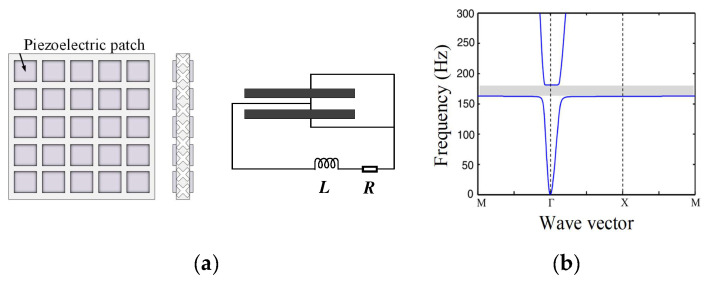
Schematics of (**a**) the piezoelectric PC with compact piezoelectric parallel array and (**b**) dispersion curves. Reprinted with permission from Ref. [58]. 2016, Chen, S.

**Figure 11:**
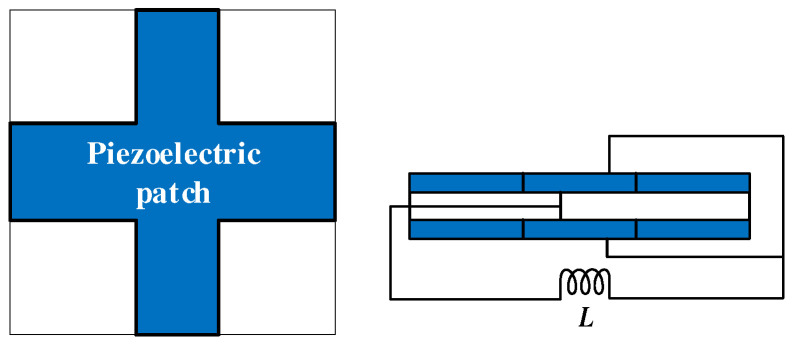
Schematic of the piezoelectric PC composed of a cross-shaped piezoelectric film. Reprinted with permission from Ref. [55]. 2018, Chen, S.

**Figure 12:**
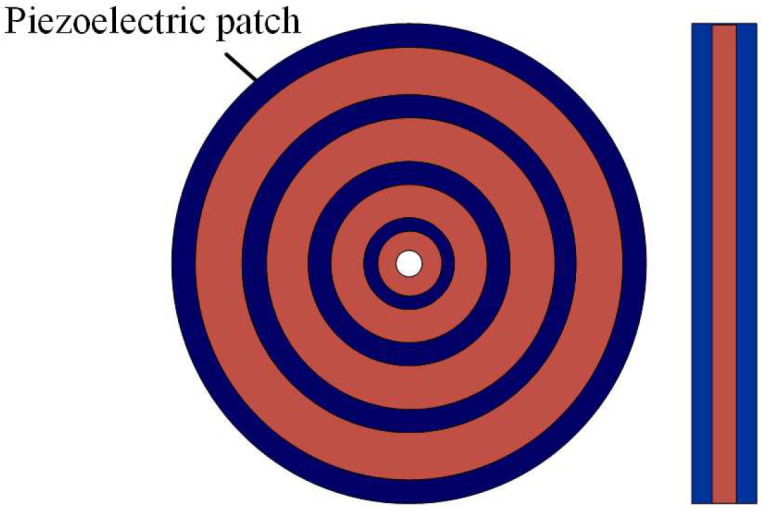
Schematic of the piezoelectric radial PC. Reprinted with permission from Ref. [59]. 2014, Shu, H.

It is always challenging to control low-frequency elastic waves, especially with broadband attenuation. LR is expected to solve this problem. However, in previous studies, piezoelectric parallel arrays are usually sparsely mounted on the substrate, which is not conducive to generating LRGs with broadband and low-frequency characteristics. Chen et al. [58] proposed a compact piezoelectric shunt array scheme, illustrated in Figure 10a. Studies have revealed that this compact structure is very efficient in the low-frequency region, and an LRG for a relative broadband can be obtained, as shown in Figure 10b. Due to the small electromechanical coupling coefficient of existing piezoelectric materials, the bandgap generated by piezoelectric shunt circuits in the low-frequency region is commonly relatively narrow. To widen the bandgap, the shape of the piezoelectric patch is a critical parameter. All piezoelectric patches in the above studies are square or rectangular, and there is a lack of research on the effect of the piezoelectric patch shape on the bandgap of PCs. Thus, Chen et al. [55] first proposed a cross-shaped piezoelectric patch with the structure shown in Figure 11. Studies have shown that the inherent capacitance of piezoelectric patches is related to their size and boundary conditions. There are fewer reports of the active control of piezoelectric radial PCs than for those with special-shaped piezoelectric films. The round plate is widely used in gearbox transmission systems, internal combustion engines, and other projects. Based on these results, Shu et al. [59] constructed a piezoelectric radial circular patch with the structure shown in Figure 12 and analyzed the influence of the structural parameters and external control gain on the propagation characteristics of flexural waves. With the increase in length ratio, thickness ratio, and period number, the bandgap will narrow and move to a low frequency, while decreasing the control gain will make the bandgap move to a low frequency. In general, the structural shape and parameters of the piezoelectric PC have a non-negligible influence on the bandgap characteristics. Higher-order vibrations and wave modalities can make the electric field inhomogeneous in such structures, which makes the commonly used uniform-electric-field assumption no longer applicable. However, the effects of the structural limitations and boundary conditions are still rarely studied. Sugino et al. [41] proposed a theoretical framework for piezoelectric PCs with double piezoelectric patches, which can generally divide these structures into multiple electrodes of any shape and study the effect of electrode shape on the response of the plate. For topology optimization and other vibration control applications, Aghakhani et al. [60] proposed a 2D piezoelectric composite plate theoretical model to analyze the influence of different pasting methods and circuit parameters on the propagation of curved waves in the frequency domain.

**Figure 13:**
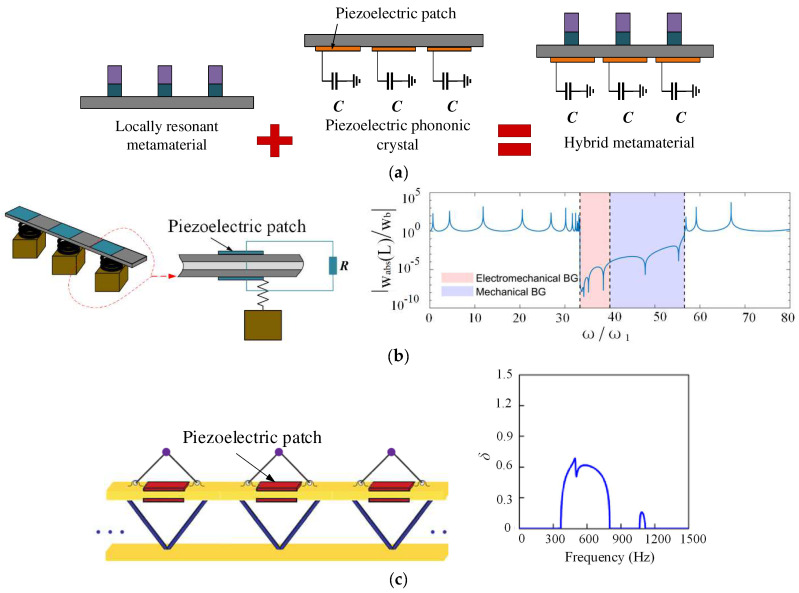
Hybrid metamaterials: (**a**) adaptive hybrid metamaterial, (**b**) a finite hybrid piezoelectric phononic crystal beam and FRF, and (**c**) the hybrid composite beam metamaterial and the attenuation diagrams [61,62,65]. Reprinted with permission from Ref. [61]. 2017, Chen, Y. Reprinted with permission from Ref. [62]. 2018, Sugino, C. Reprinted with permission from Ref. [65]. 2022, Li, J.

In addition to those studies, researchers have focused on the binding of piezoelectric PCs to mechanical metamaterials to couple the mechanical and electromechanical LR and expand the bandgap. Chen et al. [61] considered the electromechanical coupling effect and mechanical LR. They proposed an adaptive hybrid element beam that integrated NC parallel piezoelectric patches into purely mechanical LR PCs, as shown in Figure 13a. It had a negative stiffness density and a very adjustable stiffness, which indicates that the state of flexural wave propagation can essentially be changed by connecting different shunt circuits. Sugino et al. [62] introduced and analyzed mechanical–electromechanical hybrid element structures, as illustrated in Figure 13b. The hybrid superstructure demonstrated local resonant bandgaps (LRG) by utilizing mass and stiffness properties. The frequency response function (FRF) shown in the figure demonstrates that a broader bandgap is achieved when the mechanical and electromechanical excitation frequencies are in close proximity to each other. Zhou et al. [63] designed tunable PCs that consisted of an array of beams and periodic active beam resonators. An additional low-frequency and wideband bandgap were achieved by coupling the bandgaps and LRGs despite the relatively large lattice size and other structures. Liu et al. [64] also applied the hybrid concept to finite piezoelectric PC beams. Based on this result, they revealed that mechanical LRG and electromechanical LRG could simultaneously appear in a specific frequency range, which was more conducive to achieving adjustable, low-frequency, and wideband vibration reduction. Compared with the original structure, NC helps to enhance the peak attenuation of electromechanical LRG outside the bandgap coupling region. In the case of the same circuit parameters, the series method is conducive to reducing high-frequency vibration, and the parallel mode is suitable for attenuating low-frequency vibration. Previous research has mainly focused on the bandgap characteristics of hybrid structures with elastic beams or plates as the main body. Li et al. [65] conducted a study on the periodic truss sandwich beam metamaterials by employing a hybrid approach involving piezoelectric parallel array technology and the inertial amplification mechanism, as depicted in Figure 13c. The authors also presented attenuation diagrams of transverse waves within these hybrid beam metamaterials. These diagrams have proved useful in distinguishing the pass bands and stop bands by identifying the presence of zero and non-zero attenuation constants. Studies have shown that mechanisms such as electromechanical resonance, amplified inertia, and mechanical resonance can generate multiple resonant bandgaps at low frequencies, which improves the vibration suppression level. The general concept of combining mechanical and electromechanical bandgaps in the same mono-component structure is expected to be used for more complex topologies of solids and structures based on metamaterials.

In conclusion, the number and spatial dimensions of piezoelectric plates in the unit of patch-type piezoelectric PCs can be structurally designed. Compared with the other two types of piezoelectric PCs, this type has a more diversified, complex, and mature structure and external circuitry. Patch-type piezoelectric PCs have more vibration and fluctuation patterns when the dimensionality of the structure increases. However, high-order vibration and wave modes will form the non-uniform electric field of such piezoelectric structures, which makes the common assumption of a uniform electric field no longer applicable. Therefore, theoretical research to solve this problem is urgently required. The combination of piezoelectric PCs and metamaterial provides a new method to obtain ultra-wide bandgaps. The application of external circuits and active control has also improved metamaterial bandgap characteristics.

## 3. Tuning Methods of Bandgaps

**Figure 14:**
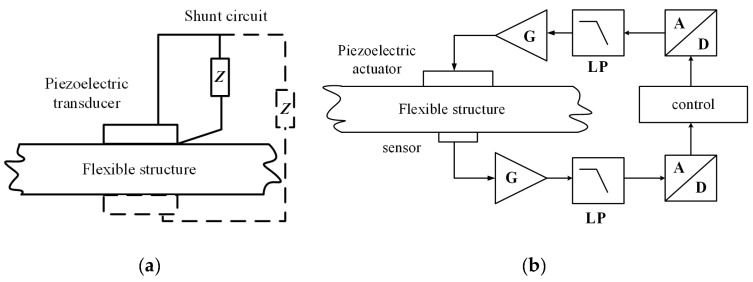
Schematics of (**a**) passive control strategy and (**b**) active control strategy. Reprinted with permission from Ref. [66]. 2018, Gripp, J.A.B.

Piezoelectric materials have been used as physical components for passive and active control systems in piezoelectric PCs to control bandgaps. Figure 14 shows their working block diagram [66]. In a passive control system, piezoelectric plates are bonded to the surface of the vibration structure as active components of inertial actuators. This includes connecting a circuit or an electronic circuit (represented by impedance Z in Figure 14a to an electrode of a piezoelectric transducer connected to the vibrating structure. Thus, the mechanical energy generated by the vibration is converted into electrical energy according to the direct piezoelectric effect, which is transmitted to the circuit and partially dissipated. The external circuits of the passive control strategy include passive, semi-passive, and semi-active circuits. In the active control system, piezoelectric sheets are used as sensors or actuators to control the mechanical vibration or fluctuation characteristics of elastic structures. The external circuits consist of charge amplifiers (G), filters (LP), and converters (A/D). The diagrams and features of relevant circuits included in these two systems are summarized in Table 1.

### 3.1. Passive Control Strategies

The passive control strategy has the advantages of low energy consumption, strong stability, and low cost. The performance of a passive control system is affected by its mechanical characteristics and external circuits. The external circuit determines the mode of power transmission and dissipation. Therefore, the design of piezoelectric parallel damping circuits will significantly affect the control effect of the structure [74,75]. Shunt circuits that do not require an external power supply (such as an R shunt circuit) are called passive circuits. Circuits that require an external power supply but do not provide any power to a mechanical structure (such as synchronized switch damping shunt circuits) are called semi-passive circuits. The R shunt circuit converts the mechanical vibration of the piezoelectric material into electrical energy in the circuit, and some of the energy is dissipated in the form of thermal energy. The resonant single-mode shunt circuit consists of resistors and inductors in series or parallel. If the circuit’s resonance is equal to that of the mechanical system, the structure exhibits a resonant behavior. This resonance characteristic causes a bandgap and will generate considerable control forces to resist the vibration of the mechanical system. Passive RL shunt circuitry can be applied to suppress the vibration of shell plates in the tunable LRG [76,77,78,79]. Nevertheless, the LRG produced by the passive RL circuitry technology is mostly narrow in piezoelectric PCs. In contrast, resonant multi-mode shunt circuits [68] can tune mechanical vibrations in more frequency bands.

A semi-active circuit indicates that it can provide power for the system through the NC shunt circuit, and the NC shunt circuit can become unstable according to the component value. NC shunt circuits [80,81] can limit the capacitance impedance of the sensor, maximize the energy dissipated by the resistance connected to the transducer, and continuously change the damping of the system in an extensive range. In addition, NC shunt circuits can effectively change the equivalent stiffness of piezoelectric materials, so they have become a research hotspot in recent years. Accordingly, when longitudinal or flexural waves propagate in structures such as plates [82] or layered [30] PCs, bandgap characteristics of PCs can be semi-passively tuned. NC shunt circuits can be simulated using a circuit that contains a constitutive component, an op-amp, a capacitor, and two resistors [70]. The series structure is more effective at suppressing low-frequency modes, while the parallel structure is more effective at suppressing high-frequency modes [83]. Adding a synthesized NC to the series RL shunt circuit increases the energy dissipation of the resonant shunt and reduces the required L. Chen et al. [84] creatively used NC shunt circuits in the piezoelectric PC, which gained a negative equivalent modulus and effectively extended the bandgap. Xu et al. [85] designed and studied dual-lane piezoelectric PC units that were connected to different NC shunt circuits, which accomplished phase differences. The destructive interference due to phase differences could enhance the elastic wave attenuation [86] and generate an extremely wide low-frequency bandgap. Tateo et al. [87] experimentally demonstrated the influence of NC shunt circuits on the propagation of flexural waves in piezoelectric composite plates. Xiao et al. [88] found that by introducing the negative capacitance into the lightweight adaptive hybrid laminate metamaterial, the bandwidth of the local resonant bandgap can be extremely enlarged. 

Changes in the external environment, temperature, and structural requirements significantly affect the regulation performance of the above shunt circuit [71,89,90]. An adaptive shunt circuit can solve this problem. A synchronous switching damping shunt circuit [72,91,92,93,94] is a semi-passive or semi-active control method that periodically turns the switch on or off according to the vibration frequency of the structure, which is equivalent to providing a nonlinear shock load. This shunt circuit does not need to accurately identify the controlled structure and can obtain better effects on vibration control and high stability. In addition, the control system is simple, and changes in the external environment have little influence.

**Figure 15:**
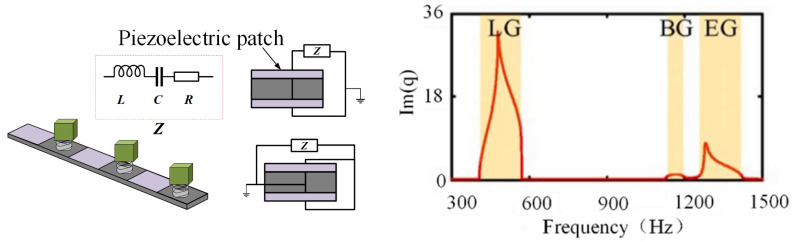
Schematic of the hybrid piezoelectric PC beam and bandgap [64].

Previous studies used uniform circuits to shunt periodic piezoelectric patches. In contrast, some researchers have turned their attention to non-uniform external circuits. Wang et al. [95] set the L of external circuits on adjacent piezoelectric patches to different values. Casadei et al. [56] connected different shunt circuits to adjacent piezoelectric patches. In both cases, the elastic structure had a different periodicity from the resonant shunt circuit. Because periodicity is not strictly required for the bandgap formation and wave attenuation, Cardella et al. [96] demonstrated that in a beam, an array of random piezoelectric elements connected by a non-uniform RL circuit could gain attenuated vibrations over a wide frequency band. However, their research was based on experiments and lacked theoretical models. The requirements for the period of PC were wholly disregarded. Despite these factors, periodic relaxation is an instructive strategy to use in the design of the tunable broadband damping of PC beams. Liu et al. [64] successfully enhanced the attenuation characteristics of finite hybrid phononic crystal (PC) beam vibration by employing a non-uniform distribution of the shunt circuit, as depicted in Figure 15. The band structure, determined by the transfer matrix method (TMM), is also presented in Figure. Notably, the frequency range exhibits the simultaneous existence of three types of bandgaps: mechanical local resonant bandgap (LRG), electromechanical LRG, and Bragg bandgap (BG). The L or C of external circuits on an adjacent unit is unevenly arranged, such as by double combination, symmetrical distribution, or gradient distribution. Through the comparison diagram of vibration transmission loss, the vibration damping performance and availability of hybrid piezoelectric PCs can be improved using the reasonable unevenly distributed parallel circuit.

### 3.2. Active Control Strategies

**Figure 16:**
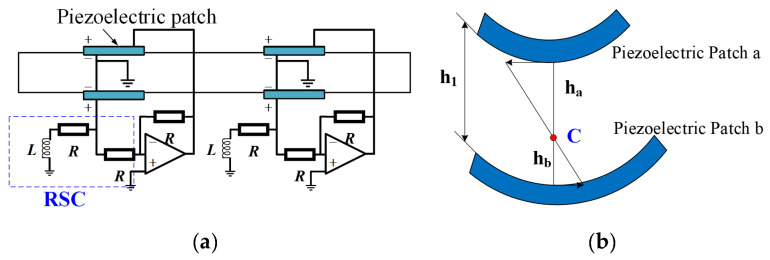
(**a**) Structure-enhanced resonant shunting circuits; (**b**) position of the bending center (C). Reprinted with permission from Ref. [95]. 2011, Wang, G.

In this subsection, the research status related to the active control of piezoelectric PCs is described. Wang et al. [95] proposed a structure containing a piezoelectric patch which is connected to the active control circuit. The voltage on the piezoelectric patch on one side of the unit is amplified by the op-amp and superimposed on the other piezoelectric patch, which enhances the resonance effect, as shown in Figure 16a. Furthermore, Wang et al. [95] calculated the Young’s modulus of the sensor and driver and considered their different effects on the flexural center (as shown in Figure 16b). The influence of the active control circuit parameters on the propagation of flexural waves was studied using the transfer-matrix method (TMM) and experiments. Using the Hamiltonian principle, Li et al. [97] equated the effects of piezoelectric plates and displacement actively controlling gain to the additional stiffness of an elastic matrix beam, but ignored the influence of the structural asymmetry. Subsequently, they studied the effect of gain detuning on the propagation of the flexural wave [98]. Chen et al. [99] and Sirota et al. [100] applied active control strategies to NC shunt circuits to control the flexural wave propagation. Li et al. [101] combined intelligent optimization algorithms with active control.

**Figure 17:**
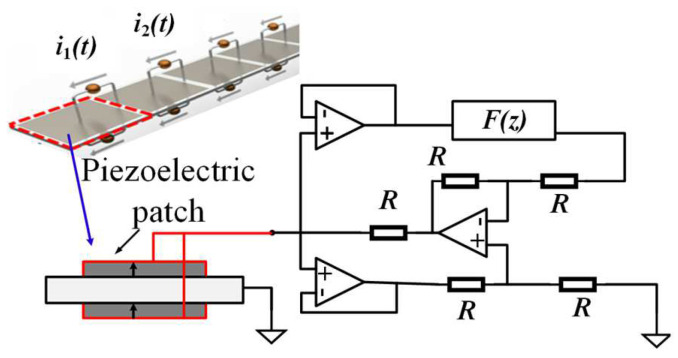
Schematic of programmable piezoelectric local resonance PCs with a digitally tunable bandgap. Reprinted with permission from Ref. [73]. 2010, Sugino, C.

In addition, digital synthetic impedance circuits are a new trend in active control. By combining a digital circuit with an NC circuit, Yi et al. [102] designed a programmable element material to control the flexural wave propagation. The transfer function in this digital circuit can be programmed in real time to place bandgaps at different target vibration patterns. Recently, Sugino et al. [73] studied a similar programmable element end bundle. It can be digitally controlled to freely adjust the impedance of the shunt circuit and bandgap of the structure, as shown in Figure 17. Subsequently, to extend the bandgap, they [103] used an electromechanical modeling framework to design and analyze piezoelectric PC beams whose units with segmented electrode pairs were shunted to synthetic impedance circuits. This method was derived from the beam theory, and it assumes that the beam is entirely covered by piezoelectric patches. Unlike the aforementioned digital circuitry, which uses the same piezoelectric patch as a sensor and actuator, Wang et al. [104] designed a digital circuit using separate piezoelectric plates as sensors and actuators. However, the derived transfer function remains limited to the beam structure and may make the resonance unstable due to the separate configuration of the sensor and actuator. Recently, Yi et al. [75] constructed a multi-resonant PC using a digital synthetic impedance circuit for bandgap adjustment on a self-sensing piezoelectric patch. The results can be used to implement any type of multi-resonant metamaterial structure, such as beams, plates, or shells.

In summary, through external circuitry or active control, unique elastic wave propagation modes can be triggered in piezoelectric PCs, which widen the Bragg scattering bandgaps, generate new LRGs, and fuse Bragg scattering and LRGs. External circuits and active control are moving toward a more diversified and integrated trend. The tuning of bandgaps for piezoelectric PCs is developing in the direction of more stability, flexibility, robustness, and efficiency.

## 4. Calculation of Bandgaps

This section introduces bandgap calculations for piezoelectric PCs on two levels: the theoretical basis of the bandgap calculation model and the calculation method. The former mainly covers the piezoelectric plate or sheet theory, electric field distribution at the electrodes, and coupling relationship between the external circuit and the piezoelectric sheet. The latter mainly includes the transfer-matrix method (TMM), plane wave expansion method (PWEM), finite element method (FEM), and spectral element method (SEM).

### 4.1. Basic Model for Bandgap Calculation

The analytical solution of the dispersion relation of the low-frequency symmetric Lamb wave of a piezoelectric plate with periodic strip electrodes can be obtained according to the piezoelectric plate theory and the assumption of a uniform distribution of the electric field between the upper and lower electrodes [105]. The resonant bandgap in the piezoelectric plate with simple external circuits can be estimated using equivalent circuits. The piezoelectric plate unit with the surface covered with electrodes can be considered an equivalent capacitor. The piezoelectric plate connected to the external circuit can be equivalent to a pure circuit. The approximate location of the resonant bandgap is predicted using the resonant frequency of the inductive capacitance circuit [26]. When the electrode length is less than the unit width of the piezoelectric plate, the equivalent capacitance of the piezoelectric plate can be obtained through numerical calculation or experiment.

Compared with single PCs, the research and analysis of patch-type piezoelectric PCs are more complicated because both piezoelectric or electromechanical coupling effects and the influence of external circuits on the elastic waves must be considered. To improve the efficiency of analysis and calculation, scholars have proposed simplified models for the theoretical or numerical calculation of the propagation characteristics of elastic waves. Among them, the most commonly used model is based on the assumption that the strain in the piezoelectric patch and electrical displacement on the electrode are equal everywhere [67]. Take the 1D piezoelectric composite rod as an example. The piezoelectric composite rod comprises a periodic piezoelectric patch and an elastic matrix rod, wherein the piezoelectric patch is connected to the RL shunt circuit. Thorp et al. [53] derived the equivalent Young’s modulus and confirmed that the effect of the shunt circuit on piezoelectric patches was to change the equivalent Young’s modulus of piezoelectric patches. Hagood et al. [67] proposed the equivalent modulus model or long-wave approximate model based on the assumption that the electric displacement on the surface of each piezoelectric patch electrode was uniform, i.e., the strain within the patch was uniform. This assumption applies to bandgaps and LRGs. Afterward, Chen et al. [106] proposed a precise integration method based on this model, and they discarded the assumption of uniform strain and obtained more general formula of electrical charges on the electrodes. The bandgaps are predicted by the application of Bloch theorem and transfer-matrix method. From a computational viewpoint, the equivalent modulus model is simpler and more efficient to implement than the precise integration model. Thorp et al. [79] extended the equivalent modulus model to 1D patch-type piezoelectric PC shells.

### 4.2. Calculation Methods

Based on the above equivalent model and piezoelectric or electromechanical coupling effect model, the researchers propose more commonly used bandgap calculation methods: the TMM, PWEM, FEM, and SEM. Table 2 shows the characteristics of these four methods.

The study of the PWEM was initiated by Wilm et al. [108], who applied the PWEM to study the propagation of elastic waves in 1D and 2D piezoelectric composite PCs. Hou et al. [109] used the PWEM to calculate the band structure of 2D piezoelectric/elastic PCs of piezoelectric scatterers or a polymer matrix. Lian et al. [110] improved the traditional plane wave unfolding method by treating the voltage as an unknown amount. For three-dimensional piezoelectric/elastic PCs, Wang et al. [111] calculated the three-dimensional surface-centered cubic piezoelectric band structure of elastic PCs. Oh et al. [34] used the FEM to construct a simulation model of a direct waveguide that consisted of a finite array of decoupled PCs surrounded by coupled PCs. The deeply tunable structure of PCs was visually illustrated. Based on the plate theory, Casadei et al. [112] introduced the influence of the shunt circuit into the governing equation of a patch in the form of the stiffness matrix and solved it using the FEM. Afterward, they connected the RL and NC circuits to the piezoelectric patch, which was periodically attached to the surface of the elastic plate, and studied the barrier effect of this combined shunt circuit on a flexural wave using the FEM. Huang et al. [113] and Lossouarn et al. [114] used the wave FEM to solve the transmission spectrum and energy band of 1D piezoelectric composite beams. Wang et al. [115] calculated the bandgap of 2D piezoelectric PCs with complex-shaped scatterers using the Petrov–Galerkin FEM. Because the FEM requires more grid elements, the calculation amount is large, and the calculation time is long. An alternative method in dynamic simulation and analysis is SEM. Several spectral elements have been developed, such as rods, beams [116,117], plates [118,119], and cables [120,121], and ongoing research proposes new and improved elements. Lee et al. [122,123] studied a spectrally formulated finite element beam with actively constrained layer damping. Afterward, Wu et al. [124] verified that SEM could be used effectively to study 3D Kagome piezoelectric grids. They also proposed and demonstrated an intelligent single-beam spectral element with passive control [125]. Recently, beam-like elements have been used to develop closed-form solutions for the dynamic behavior of lattice materials to obtain frequency-dependent representations of the effective elastic moduli [126,127].

In general, the basic model for bandgap calculation is based on the simplification of piezoelectric or electromechanical coupling effects and the equivalence of the effects on the circuit. For 2D piezoelectric PCs, the existing simplified theoretical models must be further studied and developed. No accurate simplified theoretical model has considered the influence of higher-order vibrations or waveforms on the structure of piezoelectric composite rods, beams, or plates. The development of the SEM can significantly simplify the research model of piezoelectric PCs, which provides convenience for related research.

## 5. Conclusions and Outlook

### 5.1. Conclusions

Based on the analysis of a large amount of the literature, this study aims to classify and summarize the existing research on piezoelectric PCs and clarify their primary structure, regulation mechanism, and research methods. The main conclusions are drawn as follows.

(1) Piezoelectric materials have outstanding advantages in controlling the elastic wave due to their electromechanical coupling characteristics and diversified external circuit control methods.
(2) Single and composite piezoelectric PCs have three dimensions in space. The piezoelectric material is commonly connected to the external circuit through periodic electrodes. The bandgap is predominantly controlled by periodic electrical boundary conditions. When the dimensions and types of external circuits increase, the complexity of the bandgap calculation and the variety of bandgaps also increase.
(3) Compared with the other two types of piezoelectric PCs, patch-type piezoelectric PCs have more diverse structural forms. The structural design can be conducted in terms of the number of piezoelectric patches and spatial dimensions in the unit. Patch-type piezoelectric PCs have more vibration and fluctuation patterns when the dimensionality of the structure increases. However, high-order vibration and wave modes in such piezoelectric structures can make the electric field inhomogeneous, so the uniform-electric-field assumption no longer holds. As a result, most theoretical and experimental studies on 2D patch-type piezoelectric PCs have primarily been limited to elastic patches and low or ultra-low frequency. The combination of piezoelectric PCs and mechanical superstructures provides a new method to achieve ultra-wide bandgaps. Using an external circuit or active control, a special elastic wave propagation mode can be induced to widen the bandgaps. In other words, new LRGs will be generated, and Bragg scattering can be combined with LR.
(4) The development of FEM and SEM has promoted the visualization and simplification of bandgap tuning for piezoelectric PCs.

### 5.2. Outlook

The following are future research directions concerning single and composite piezoelectric PCs. First, special microstructures such as resonant units or related concepts (including topological acoustics) should be explored further to promote the integrated design. Second, there is a lack of attention to the structural parameters and inherent shape of piezoelectric PCs; therefore, future studies should focus on optimizing the topology to improve the acoustic and vibration characteristics. Third, the coupling of complex external circuits should be studied, which has been ignored in most previous studies. Fourth, the method to break the symmetry of the structure, e.g., by changing the electric field boundary conditions of the upper and lower electrodes, to achieve the LRG of the anti-symmetric Lamb wave mode should be improved in the future.

The higher dimensionality of the structure in the patch-type piezoelectric PC enables it to have more vibrational and wave modes. However, high-order vibration and wave modes will make the electric field in such piezoelectric structures inhomogeneous, which makes the commonly used assumption of a uniform electric field no longer applicable and considerably limits its application and research. The widely used research model is based on the simplified decoupling of an elastic force field and an electric field, and its application scope requires further research and development. In particular, the precise simplified theoretical model that considers the effects of higher-order vibration or wave modes of the structure remains insufficient. Nonetheless, such models can simplify complex analyses and improve the computational efficiency. The combination of patch-type piezoelectric PCs and mechanical superstructures provides a new method to achieve an ultra-wide bandgap that deserves attention. The existing related research models are based on simplifying the decoupling of the elastic force field and electric field. For 2D structures, there is currently no accurate simplified theoretical model that considers the influence of the high-order vibration or wave modes of the structure. However, such models can simplify complex analyses and improve the computational efficiency.

In addition, it is necessary to quickly and accurately develop equipment that can process mechanical signals to make full use of the positive/negative piezoelectric effects. External control circuits with better performance (such as good stability, accurate adjustment, wide range, small volume, and low energy consumption) must also be designed. Integrating machine learning, deep learning, neural network, and other artificial intelligence concepts into the control of external circuits is a promising research direction. Existing related research and corresponding experimental verification research are relatively scarce and urgently need enhancement. The rapid development of advanced 3D/4D printing technology and the new generations of vibration and wave testing equipment have provided opportunities for relevant experimental research.

## Figures and Tables

**Table 1:** Summary of common piezoelectric shunt circuits. Reprinted with permission from Ref. [66]. 2018, Gripp, J.A.B.

Circuit	Circuit Diagram	Advantage	Disadvantage
R shunt [67]	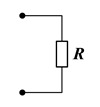	It has a passive character, and its structure is simple and low cost. It can also dissipate the vibration energy of the structure and suppress the vibration without electromagnetic vibrations.	It can only generate bandgaps, not LRGs.
Resonant single-mode shunt [67]	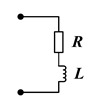	It has a passive character, and its simple structure (in series or parallel) enables the formation of LRGs.	The effective vibration damping range is narrow, requires a large L at low frequencies, and produces electromagnetic oscillations.
Resonant multi-mode shunt [68]	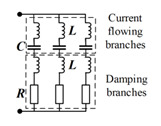	It has a passive character and is effective in multiple frequency bands compared with the single-mode shunt circuit.	When the number of resonant modes increases, the structure becomes more complex and expensive.
NC shunt [69,70]	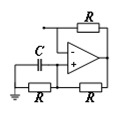	It has a semi-passive character and can continuously adjust large frequency bands. It can generate both high- and low-frequency bandgaps. It can be combined with other resonant circuits.	Its structure is relatively complex, the performance is unstable, and the cost is high.
Adaptive shunt [71]	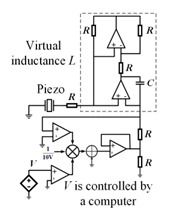	It is often combined with different shunt circuits. Its impedance can be adaptively adjusted online to adapt to the change in structural resonance frequency caused by the change in the external environment or demand. In addition, it can adapt to the change in capacitance of the piezoelectric sensor caused by the change in external temperature.	This circuit is very complex and bulky in practical application, and it requires an external personal computer to run the adaptive algorithm.
Switch shunt [72]	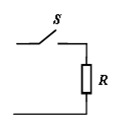	It has semi-passive or semi-active and nonlinear characters. The circuit switch is synchronous with the vibration period of the structure and can adapt to different excitation frequencies.	It will excite high-order harmonics from the pulse voltage inversion. For structures with effective acoustic radiation, series inductors in the circuit are not suitable for acoustic driving.
Non-uniform [56]	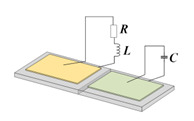	It has the characteristics of non-periodicity. It can break the symmetry property and make it possible to obtain unique and favorable phenomena. A wider bandgap can be produced.	It remains in the preliminary stage and lacks a powerful theoretical model.
Digital [73]	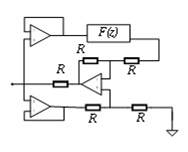	It is active and can be fed according to the transfer function realized by a digital signal processor. The transfer function in this digital circuit can be programmed in real time to set bandgaps at the location of different target vibration patterns.	The separate configuration of sensors and actuators will make the resonance unstable. Its complex structure and limited application scope of the transfer function significantly limit its practical application.

**Table 2:** Summary of common piezoelectric PC research methods.

Method	Characteristics
TMM [107]	This method is suitable for structures where the boundaries are ignored or treated as periodic boundaries, and the system can be decomposed into subsystems where only adjacent elements interact. It is often used to solve the bandgap characteristics of 1D PCs and finite periodic structures, and the computational cost is low.
PWEM [108,109,110,111]	This method is mainly used to calculate the bandgap of PC containing solid and solid, liquid and liquid, or solid and gas interactions. It is commonly used to solve 2D/3D PCs. However, the bandgap control characteristics of PCs containing liquid, gas, and solid cannot be accurately calculated. When the material parameters of PC components are different, the computational burden is enormous, and the convergence speed is slow.
FEM [34,112,113,114,115]	This method has a fast calculation speed and good convergence, so it can accurately calculate the characteristics of PCs of various complex structures and intuitively display them. However, boundary conditions are difficult to determine and often not sufficiently precise. In addition, the method has more requirements for grid elements, a large amount of calculation, and a long calculation time. Several large-scale commercial FEM software, such as ANSYS and COMSOL Multiphysics, are commonly used.
SEM [116,117,118,119,120,121,122,123,124,125,126,127]	This method has the advantages of both wave propagation analysis and finite element analysis and dramatically reduces the number of simulation elements. A single element is sufficient to simulate smart materials with uniform cross-sections along the length. It has been effectively used to analyze the dynamic response and wave propagation characteristics of piezoelectric PC beams. However, the Gibbs phenomenon will appear in the model with complex geometry and a discontinuous interface, which seriously affects the accuracy of the model.

## Data Availability

Not applicable.
